# CT-Guided Online Adaptive Stereotactic Body Radiotherapy (SBRT) for Retroperitoneal Sarcoma After Previous Radiotherapy: A Case Report

**DOI:** 10.7759/cureus.69239

**Published:** 2024-09-12

**Authors:** Adham Hijab, Yonina Tova, Shlomi Alani, Sharon Amitzur

**Affiliations:** 1 Radiotherapy, Ziv Medical Centre, Safed, ISR

**Keywords:** online-adaptive radiotherapy, radiotherapy (rt), re-irradiation, sarcoma, stereotactic ablative body radiotherapy

## Abstract

CT-guided online adaptive radiotherapy (OART) is a novel and robust treatment technique in radiotherapy. Thanks to its excellent accommodation of inter-fraction variations, OART is characterized by superior accuracy compared to other contemporary treatment techniques in radiotherapy such as image-guided radiotherapy (IGRT). Planning target volume (PTV), which takes into account interfraction movements, could therefore be reduced while utilizing OART with a consequent dose reduction for adjacent healthy tissue.

Herein we report our successful experience in treating a patient with retroperitoneal sarcoma after previous radiotherapy and surgery. The tumor was in close proximity to the spinal canal and abutted a large bowel segment and the last portions of the duodenum. Radiotherapy regimen consisted of 30 Gy in five fractions. Treatment implementation and delivery were feasible and the treatment was given without any interruptions.

After a follow-up period of nine months so far, the patient reported no radiation-related adverse effects. Furthermore, her post-treatment magnetic resonance imaging (MRI) scans demonstrate good radiographic response.

Our case highlights the use of OART to treat a challenging case in radiotherapy as it was given in the setting of re-irradiation to a mesenchymal tumor, which is considered exquisitely radio-resistant.

## Introduction

Online adaptive radiotherapy (OART) is an emerging, revolutionary treatment technique in radiation oncology. One of the two commercially available platforms of OART is the Ethos system (Varian Medical System, Palo Alto, CA). The Ethos OART platform generates adapted radiation plans prior to each fraction based on computerized topography (CT), which is acquisitioned while the patient is on the treatment couch, i.e., cone beam CT (CBCT). Following CBCT acquisition, contours are regenerated with the support of an artificial intelligence (AI) system while other target volumes may undergo deformable propagation. What results is a newly calculated radiation plan before each fraction. This excellent accommodation to inter-fraction variations significantly diminishes uncertainties in radiation plans. This, in turn, enables the clinicians to safely reduce the planning margins around the target volume, hence avoiding unwanted exposure of adjacent organs at risk (OARs) to significant radiation doses.

Several studies have demonstrated both improved dosimetry of OARS and reduced toxicity with the use of OART compared to image-guided radiation therapy (IGRT) [1-3].

The augmented therapeutic window associated with OART would also enable clinicians to treat cases that were once considered challenging by the “conventional radiotherapy” dogma. Delivering ablative radiation doses to sites which were previously irradiated with high radiation doses would be one example of such cases.

Herein we present a case in which we leveraged OART to re-irradiate recurrent retroperitoneal sarcoma which abutted critical organs that were previously exposed to high doses of radiation.

## Case presentation

**Figure 1:**
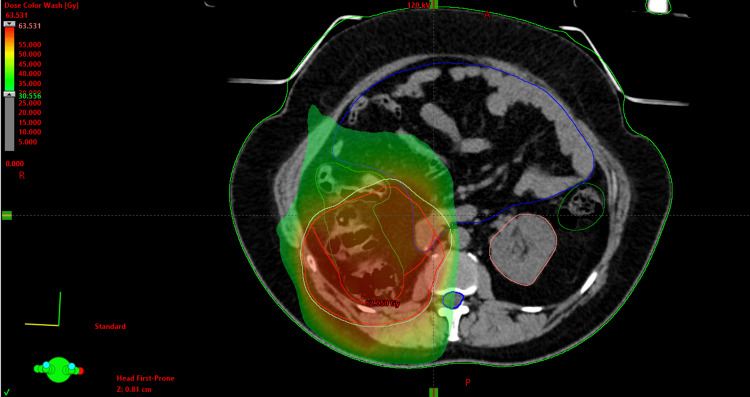
Representative axial CT slice of adjuvant radiotherapy plan, which the patient received after her first surgery. The tumor bed received up to 60 Gy. The maximal point dose received by the large bowel, duodenum, and spinal canal were 62.36, 62.38, and 50.24 Gy, respectively

A 57-year-old otherwise healthy female patient was initially diagnosed with retroperitoneal sarcoma in November 2020. At that time, she underwent frontline extended surgery along with radical right nephrectomy and adrenalectomy. Pathology revealed a high-grade spinde cell sarcoma with a KI67 around 40%. The tumor measured 4 cm in maximal diameter and had inconclusive resected margin status. The patient went on to receive adjuvant radiotherapy, which constituted of 50 Gy in 25 fractions, followed by a sequential boost of 10 Gy in five fractions to the paravertebral space, which corresponded to the tumor bed (Figure 1). She tolerated radiation treatment well and she continued follow-up. 

**Figure 2:**
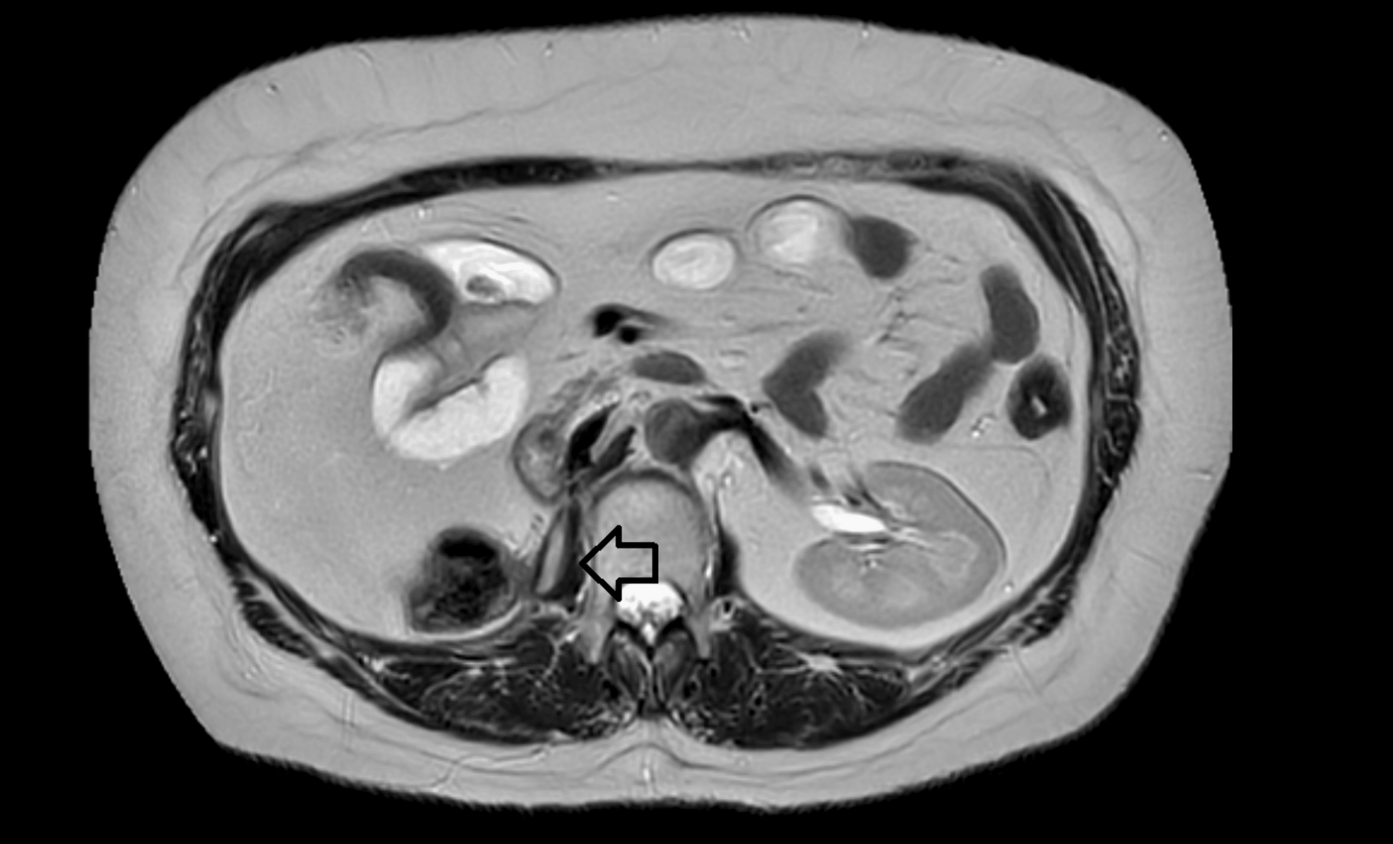
An axial slice of T2-weighted MRI image demonstrating disease recurrence (arrow) in the right psoas muscle with the lesion exhibiting hyperintense signal

In October 2022, a local recurrence, adjacent to the right psoas muscle, was documented (Figure 2) and the patient had a second surgery in April 2023. Pathology revealed a completely resected 5X8X9 cm mass, which was classified as low-grade dedifferentiated liposarcoma.

**Figure 3:**
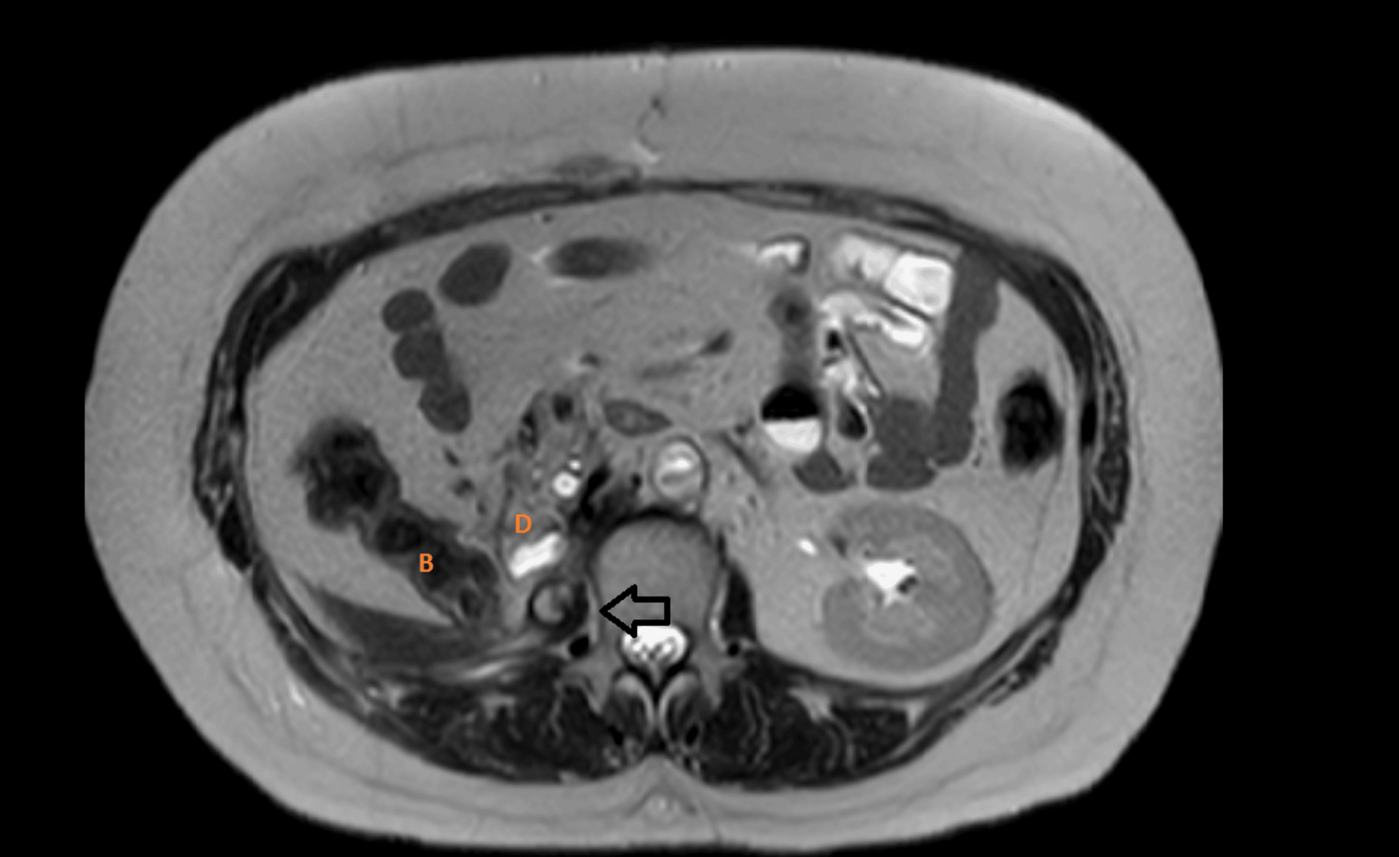
T2-weighted axial MRI image showing a hyperintense lesion (arrow) in the right psoas abutting the duodenum (D) and large bowel (B).

However, a magnetic resonance imaging (MRI) scan in August 2023 revealed a second event of local recurrence in the right psoas muscle as shown in Figure 3. Surgery at this point was deemed not feasible and she was referred to our department in order to consider another course of radiotherapy.

Clinically, the patient felt well and was asymptomatic. She recovered very well from her surgery and had an uneventful post-operative course. After reviewing her imaging scans, we could determine the following: firstly, the suspected lesion had radiographic characteristics that were highly suspicious for malignancy (moderately hyperintense in T2-weighted image, diffusion restriction in diffusion-weighted image, and prominent gadolinium enhancement). Secondly, we noticed a very close proximity between the lesion and the distal parts of the duodenum (third and fourth parts), the proximal part of the jejunum and a large bowel segment. Thirdly, after reviewing various scans across a broad timeline after her first surgery, we noticed that there were almost no variations in terms of spatial relations between the lesion and the aforementioned bowel segments and this could be due to abdominal adhesions (secondary to surgery), which would restrict bowel loops mobility within the peritoneal bag. 

Therefore, we decided to pursue an ultra-hypofractionated course of radiotherapy utilizing the OART capabilities of the Ethos platform. The patient underwent a contrast-enhanced planning CT with a slice thickness of 1mm in the supine position. Knee and foot locks were used to ensure stable position. Prior to CT acquisition, the patient was given 50 mL oral contrast to better visualize the bowel loops. 

The gross tumor volume (GTV) was delineated with the aid of an experienced radiologist and all available diagnostic scans. No marginal expansions were applied around the GTV. The bowel bag, duodenum, spinal canal, and the sole remnant kidney were also delineated as OARs. Of note, a planning risk volume (PRV) was generated around the spinal canal by adding an isotropic 2-mm margins. 

We prescribed to the GTV 30 Gy given in five fractions, given every other day. The coverage objectives and OARs dose constraints are presented in Table 1. 

**Table 1:** Target volume goals and OARs constraints based on Dx, which is the percentage of structure volume or absolute volume in cm3 that receives the minimal (Dx ≥ Y Gy) or maximal (Dx ≤ Y Gy) assigned dose. OAR: organ at risk; GTV: gross tumor volume; PRV: planning organ at risk volume

Volume	Goal
GTV	D99% ≥ 28.5 Gy
	D0.3% ≤ 32.1 Gy
Bowel	D0.1cm^3^≤ 30Gy
	D5cm^3^≤ 17.7Gy
	D10cm^3^≤ 25Gy
Duodenum	D0.1cm^3^≤ 33Gy
	D10cm^3^≤ 25Gy
PRV_spine	D0.03cm^3^≤ 20.3 Gy

**Figure 4:**
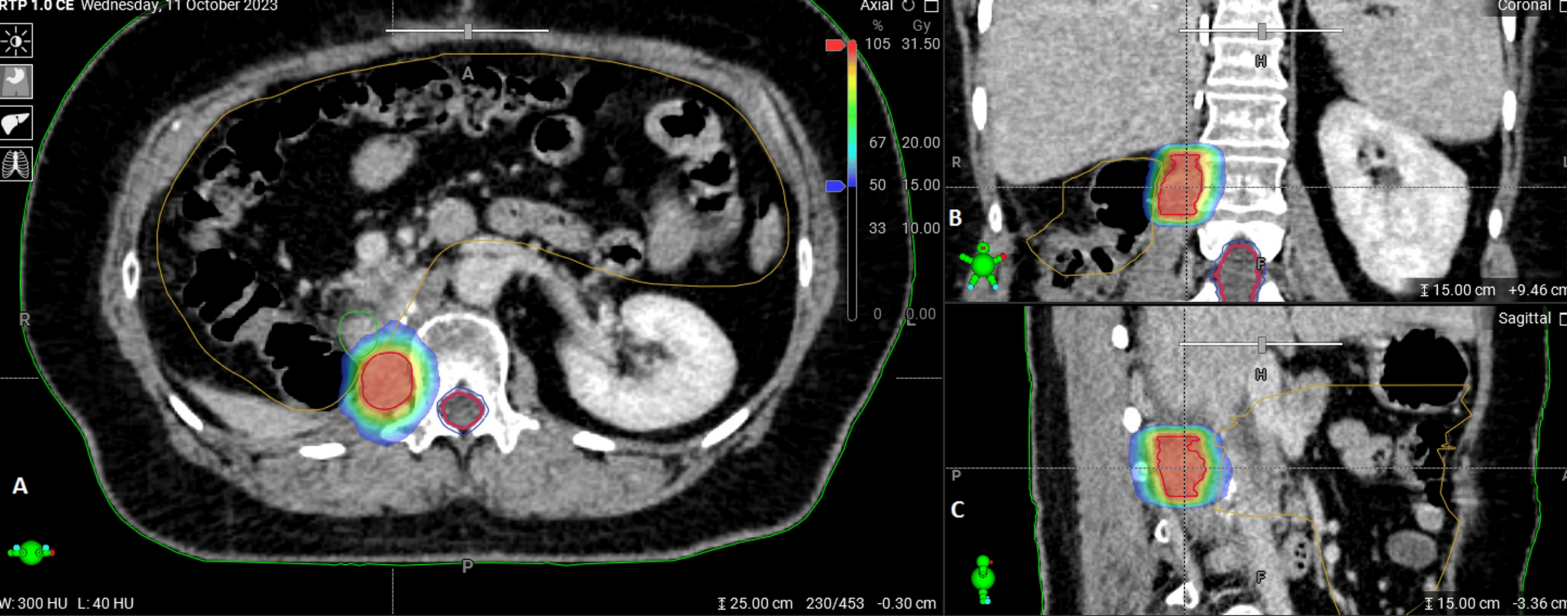
The calculated re-irradiation plan based on the Ethos treatment planning system presented in axial (A), coronal (B), and sagittal (C) planes. The 50% dose fall-off gradient was within 1.45 cm from the PTV, which corresponded to GTV as no planning margins were applied. PTV: planning target volume; GTV: gross tumor volume

A 12-fields Intensity-modulated radiation therapy (IMRT) plan (Figure 4) was selected out of three generated IMRT plans (seven, nine, and 12 fields). 

The patient received treatment as scheduled. The average total treatment time (from patient entering the treatment room until the end of radiation delivery) was 27 minutes and 44 seconds. The average time between the first CBCT and plan selection was 10 minutes and 26 seconds. The average time between plan selection and end of radiation treatment was 8 minutes and 38 seconds. The adapted plan was selected for treatment delivery in all five fractions given significantly better dose coverage of the target volume. On average, the D99% of GTV obtained in the scheduled plan was 24 Gy (80.26% of the prescription dose) compared to 28.48 Gy in the adapted plan (94.96% of the prescription dose). Of note, OARs dose constraints were fulfilled both in the scheduled and the adapted plan with nonsignificant, favorable dosimetry in the adapted plan. 

**Figure 5:**
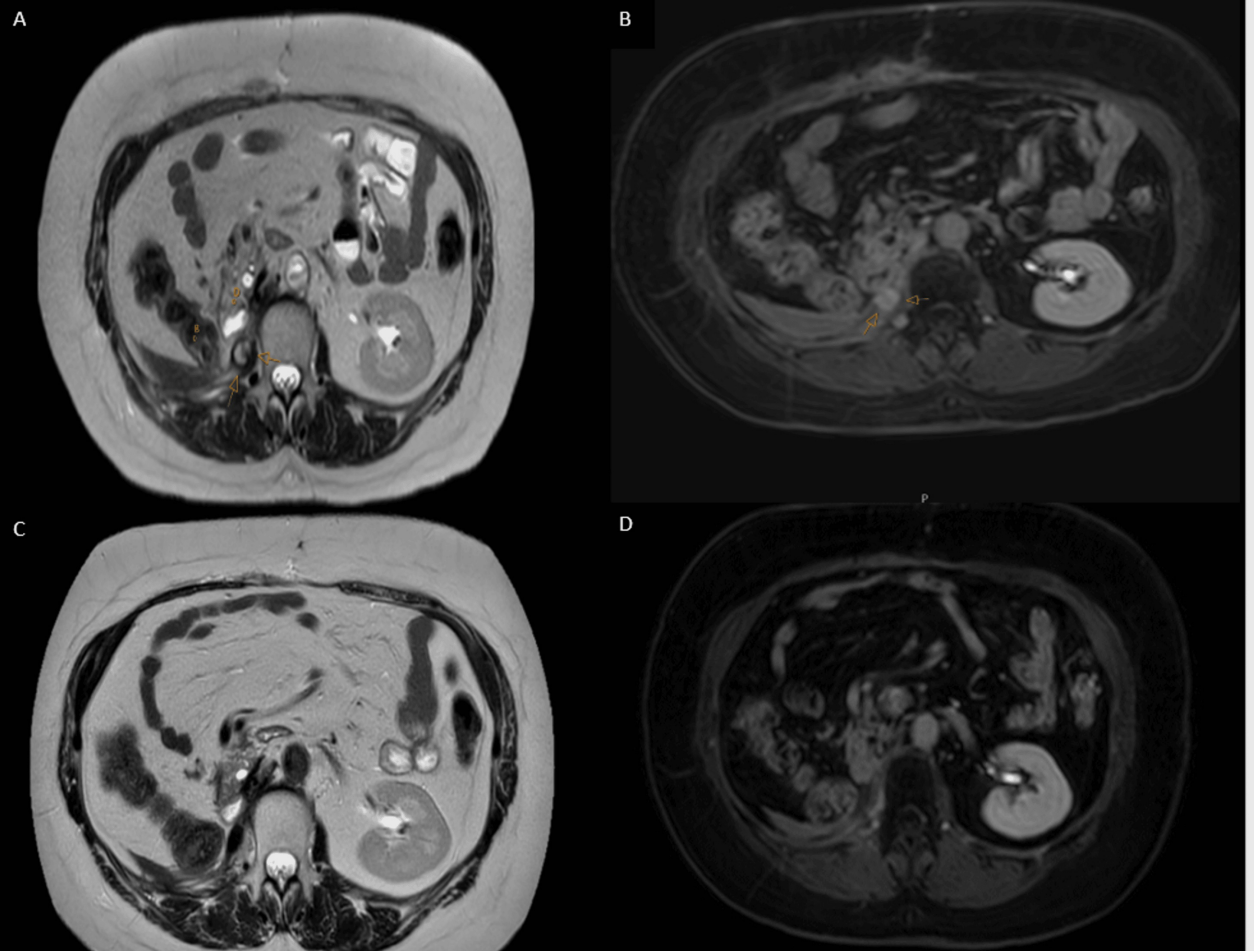
Radiographic response post-radiotherapy. The hyperintense signal seen in T2-weighted image (A) has resoluted (C). In addition, the prominent gadolinium enhancement (B) has significantly diminished (D).

After nine months of follow-up post her second course of radiotherapy, the patient feels well and without any symptoms that could be attributed to radiation treatment. She had two MRI scans following her radiotherapy, which showed complete response in right psoas. She continues follow-up without any active treatment. Figure 5 illustrates a comparison between sectional images of her MRI scans prior and after radiation therapy.

## Discussion

The robust accuracy of OART facilitates the exploration of new avenues in radiotherapy, with the hope of eventually increasing treatment efficacy without elevating the risk of toxicities. Many large-scale clinical trials are currently ongoing with promising initial results, which highlight the advantage of OART. In addition, many groups have reported their experience with OART in various disease sites [1-8].

Treating radio-resistant tumors (such as the majority of sarcoma’s subgroups) in sensitive areas like in the abdomen or pelvis could be feasible thanks to the excellent conformality that could be achieved with OART. Our case demonstrated this possibility when surgery was deemed not feasible and radiotherapy was the only possible local treatment. This case was also complicated by the fact that the patient had already a radical dose of radiotherapy delivered to that area only few years before and that critical structures such as the spinal canal and bowel have already reached the upper threshold of acceptable dose constraints. The average total session duration was reasonable and the actual time in which the patient spent on the treatment couch was less than 20 minutes on average. While OART provides better accuracy by adjusting to inter-fractional variations, this come with the cost of a prolonged radiotherapy session, which may give way to intra-fractional variations that may necessitate the restart of the whole treatment session (such as the emergence of a prominent air bubble in bowel or rectum). Moreover, prolonged treatment time can increase patient discomfort during treatment as some patients may struggle to adhere to the planning setup for a considerable time. Therefore, using OART should be done in a timely manner in order to overcome the aforementioned downsides. 

Despite the advantages associated with OART, it has several drawbacks that should be acknowledged. Firstly, the workflow of OART is significantly different from conventional radiotherapy treatment. While treating a patient with OART, both physician and medical physicist should be present at least in part of the treatment in order to validate the regenerated structures and to sign off the newly calculated plan. Secondly, treatment duration is also significantly longer compared to IGRT treatment due to the additional steps involving contour review, plan generation, and plan approval. These factors could limit the wide implementation of OART due to capacity issues and limited workforce in some of the radiotherapy departments [9]. Thirdly, visualization of volumes in CT-guided OART could be hampered by the relatively poor soft-tissue contrast resolution of CBCT, rendering some sites and diseases particularly challenging to treat. 

Using OART in our case enabled us to adequately cover the target volume while fully respecting the dose constraints that we determined. For extra caution, we applied no margins around the GTV and we achieved excellent outcomes regardless of that, which again proves the excellent accuracy associated with OART system.

## Conclusions

OART is a valuable tool that could be leveraged to successfully treat intricate cases such as in the setting of re-irradiation and delivering ablative doses to radio-resistant tumors. Re-irradiation of recurrent retroperitoneal sarcoma is feasible with reassuring outcomes both in terms of toxicity and efficacy. We believe that one of the main advantages of OART would be in the context of salvage radiotherapy after previous, radical doses of radiotherapy. 
